# Thermal adaptation of mesophilic and thermophilic FtsZ assembly by modulation of the critical concentration

**DOI:** 10.1371/journal.pone.0185707

**Published:** 2017-10-05

**Authors:** Luis Concha-Marambio, Paula Maldonado, Rosalba Lagos, Octavio Monasterio, Felipe Montecinos-Franjola

**Affiliations:** Laboratorio de Biologia Estructural y Molecular/Departamento de Biologia/Facultad de Ciencias, Universidad de Chile, Santiago, Chile; Consejo Superior de Investigaciones Cientificas, SPAIN

## Abstract

Cytokinesis is the last stage in the cell cycle. In prokaryotes, the protein FtsZ guides cell constriction by assembling into a contractile ring-shaped structure termed the Z-ring. Constriction of the Z-ring is driven by the GTPase activity of FtsZ that overcomes the energetic barrier between two protein conformations having different propensities to assemble into polymers. FtsZ is found in psychrophilic, mesophilic and thermophilic organisms thereby functioning at temperatures ranging from subzero to >100°C. To gain insight into the functional adaptations enabling assembly of FtsZ in distinct environmental conditions, we analyzed the energetics of FtsZ function from mesophilic *Escherichia coli* in comparison with FtsZ from thermophilic *Methanocaldococcus jannaschii*. Presumably, the assembly may be similarly modulated by temperature for both FtsZ orthologs. The temperature dependence of the first-order rates of nucleotide hydrolysis and of polymer disassembly, indicated an entropy-driven destabilization of the FtsZ-GTP intermediate. This destabilization was true for both mesophilic and thermophilic FtsZ, reflecting a conserved mechanism of disassembly. From the temperature dependence of the critical concentrations for polymerization, we detected a change of opposite sign in the heat capacity, that was partially explained by the specific changes in the solvent-accessible surface area between the free and polymerized states of FtsZ. At the physiological temperature, the assembly of both FtsZ orthologs was found to be driven by a small positive entropy. In contrast, the assembly occurred with a negative enthalpy for mesophilic FtsZ and with a positive enthalpy for thermophilic FtsZ. Notably, the assembly of both FtsZ orthologs is characterized by a critical concentration of similar value (1–2 μM) at the environmental temperatures of their host organisms. These findings suggest a simple but robust mechanism of adaptation of FtsZ, previously shown for eukaryotic tubulin, by adjustment of the critical concentration for polymerization.

## Introduction

Cytokinesis is the last stage in the cell cycle. In most prokaryotic cells, cytokinesis is driven by contraction of the Z-ring, an intracellular polymer assembled by the essential protein FtsZ [1]. FtsZ is a well conserved protein found in Bacteria (in all phyla with few exceptions), in Archaea (exclusively in the phylum Euryarchaeota), and in Eukarya (in intracellular organelles such as chloroplasts and mitochondria) [2, 3]. Many details about prokaryotic cytokinesis have been elucidated from the study of mesophilic bacterial models, such as the gram negative proteobacterium *Escherichia coli* or the gram positive firmicute *Bacillus subtillis* [4–6]. By contrast, few studies have directly studied Archaeal cytokinesis. For instance, the presence of FtsZ homologues in members of the archaeal phylum Euryarchaeota, which are often extremophiles, suggest a mechanism of division similar to that of Bacteria [7]. In the other major archaeal phylum Crenarchaeota, the thermophilic genus *Sulfolobus* lacks FtsZ but cytokinesis is supported by the Cdv machinery that works by a mechanism similar to that of the Z-ring, i.e., by polymerizing into a ring-shaped structure at the site of cell division [8, 9]. The Cdv proteins are homologous with components of the eukaryotic endosomal sorting complex required for transport (ESCRT), which participates in membrane bending and scission functions, including cytokinesis [10]. In mesophilic *Nitrosopumilus maritimus*, a member of archaeal phylum Thaumarcheaota, both FtsZ and the Cdv proteins are found but only the latter has been proposed to work in cell division [11, 12]. Thus, prokaryotic cell division functions through a combined mechanism of protein-condensation followed by a constriction of the cell membrane, regardless of the factors involved or the environmental temperature.

The protein FtsZ is related to other proteins of similar tridimensional structure found in the FtsZ/Tubulin family, whose members share the ability to bind GTP and assemble into linear polymers with shapes similar to the protofilaments formed by eukaryotic tubulin [13–15]. The members of the FtsZ/Tubulin family are involved in cell division, intracellular trafficking, chromosome segregation, or in controlling cell shape. A comparison between the structures of dimers belonging to the FtsZ/Tubulin family reveals an analogous packing of the subunits indicating a similar cooperative mechanism in formation of protofilaments [15–17]. The two best characterized FtsZ are those from mesophilic *Escherichia coli* (EcFtsZ, optimal growth at T = 37°C), and from thermophilic *Methanocaldococcus jannaschii* (MjFtsZ, optimal growth at T = 85°C). Studies on the polymerization kinetics of FtsZ have shown that the critical concentration (C_C_) for the GTP-induced polymerization is around 1 μM [18–20]. FtsZ polymerizes mainly into linear filaments, but may also assemble into bundles, sheets and rings, depending on the experimental conditions [21–23]. The hydrolysis of GTP modulates the dynamics of FtsZ assembly, by serving as an energetic barrier between two conformations having different polymerization affinities [20, 24–28]. This change in conformation of FtsZ is thought to be the driving force for contraction of the Z-ring. For these reasons, understanding the energetics of FtsZ polymerization is key for describing the fundamental steps controlling prokaryotic cytokinesis.

While the energetics of thermophilic MjFtsZ has been investigated before, a systematic study of the temperature dependency of mesophilic FtsZ assembly is lacking. In this work, we measured the GTP hydrolysis and polymerization kinetics of mesophilic FtsZ in comparison with thermophilic MjFtsZ, in a temperature range close to their normal growth temperature. In other words, we addressed the question of whether prokaryotic cell constriction may operate similarly at moderate (mesophilic) temperatures compared to higher (thermophilic) temperatures. We measured the GTPase activity and the polymerization kinetics of FtsZ under saturating substrate concentrations, ensuring maximal rates of nucleotide hydrolysis and steady-state polymerization conditions. While our data indicated similar stabilities for the FtsZ-GTP transition state complex for both mesophilic and thermophilic FtsZ, in contrast, we observed enthalpically-driven assembly for mesophilic EcFtsZ and entropically-driven assembly for thermophilic MjFtsZ. These findings are discussed in the context of the proposed mechanism for FtsZ polymerization and in comparison, with the previously described mechanism of tubulin assembly. Our data suggest a conserved mechanism of adaptation of FtsZ assembly to different environmental temperatures by modulation of the critical concentration.

## Materials and methods

### Protein purification and quantitation

Wild type *Escherichia coli* FtsZ (mesophilic EcFtsZ), was overexpressed in *E. coli* BL21 (DE3) and purified using polymerization-depolymerization cycles as previously described [29]. *Methanocaldococcus jannaschii* FtsZ-his_6_ fusion protein (thermophilic MjFtsZ), was overexpressed in exponentially growing *E. coli* BL21 (DE3) using 0.4 mM IPTG. After 3 hours of induction the bacterial culture was harvested by centrifugation, suspended in A buffer (50 mM Tris-Cl pH 8.0, 300 mM KCl and 10% glycerol), and supplemented with EDTA free protease inhibitor (Roche, Branchburg, NJ). Then, the cell suspension was lysed by sonication and centrifuged at 100,000 x g at 4°C for 1 hr. The supernatant was adjusted to 5 mM imidazole and then loaded onto a Ni-Sepharose affinity column (GE Healthcare, Piscataway, NJ). Elution was carried out at 1 ml/min using a linear gradient of imidazole, all at 4°C, and MjFtsZ eluted at 500 mM imidazole. The purified protein fractions were dialyzed against cold A buffer, concentrated using Amicon Ultra filters (EMD Millipore, Billerica, MA), and stored at -80°C. Coomassie blue stained SDS PAGE densitometry analysis showed at least 95% purity for both protein preparations. The protein concentration was determined spectrophotometrically in 6 M GdmCl phosphate buffer assuming 1 molecule of GDP bound per protein monomer. The extinction coefficients employed for the apoproteins were: EcFtsZ ϵ_280nm_ = 3,840 M^−1^cm^−1^ and ϵ_254nm_ = 2,750 M^−1^cm^−1^, for MjFtsZ ϵ_280nm_ = 6,970 M^−1^cm^−1^ and ϵ_254nm_ = 4,275 M^−1^cm^−1^, and for the GDP nucleotide: ϵ_280nm_ = 8,100 M^−1^cm^−1^ and ϵ_254nm_ = 13,620 M^−1^cm^−1^.

### FtsZ GTPase activity

GTP hydrolysis was measured by quantifying the released inorganic phosphate using the malachite green colorimetric method. Mesophilic EcFtsZ was equilibrated at the indicated temperatures in 50 mM MES-KOH pH 6.5, 5 mM MgCl_2_ and 50 mM KCl. In the case of thermophilic MjFtsZ, buffer composition was adjusted to 300 mM KCl to maintain consistency with the storage buffer, which in our experience improved the solubility of the protein. The temperature was controlled to ± 0.2°C using a water bath. A small temperature probe (model BAT8, Bailey Instruments, Saddle Brook, NJ) was used to check the temperature *in situ*. The polymerization reaction was started by the addition of 1 mM GTP. At desired times, a 30 μl aliquot of the reaction mixture was added to 770 μl of cold 0.32 M perchloric acid to stop the reaction. Then, a 160 μl aliquot of this mixture was deposited in triplicates onto 96-well plastic plates, followed by addition of 40 μl of the dye mixture to each well. The dye mixture consisted of: 0.77 volumes (vol) of 1.32 M malachite green dissolved in H_2_SO_4_ 3.1 M, 0.19 vol of 15% ammonium heptamolybdate dissolved in water and 0.04 vol of 11% v/v aqueous Tween 20. The plates were incubated at room temperature for 30 minutes in the dark. Then, the absorbance at 630 nm was measured in an Epoch ELISA reader (Biotek Instruments Inc, Winooski, VT). The phosphate concentration was determined by interpolation on a 12-point calibration curve prepared with KH_2_PO_4_ in the range between 0 and 450 μM, which was loaded in the same 96–well plate containing the samples of interest.

The production of inorganic phosphate P_i_ due to the hydrolysis of GTP can be described by the following scheme (Eq 1):

$$\begin{array}{c} \text{FtsZ}\cdot \text{GDP}+\text{GTP}\overset{k_{+1}}{\underset{k_{-1}}{⇌}}\text{FtsZ}\cdot \text{GTP}+\text{GDP}\overset{k_{cat}}{\to }\text{FtsZ}\cdot \text{GDP}+\text{GDP}+{\text{P}}_{\text{i}} \end{array}$$ (1)

where *K*_GTP_ = *k*_–1_/*k*_+1_ is the FtsZ nucleotide exchange equilibrium constant and *k_cat_* is the first order hydrolysis rate constant (or catalytic constant). At saturating GTP concentrations, the exchange reaction quickly reaches equilibrium, and the nucleotide hydrolysis is the rate-limiting step of the reaction such that *k_cat_* ≫ *k*_–1_. To avoid the interference of the accumulated GDP in the assembly reaction [30], we measured the initial rates of GTP hydrolysis by collecting data during the first 4-6 minutes after starting the reaction. The observed initial rate of GTP hydrolysis *k_obs_* is given by the first-order rate law (Eq 2):

$$\begin{array}{ccc} k_{obs} & = & k_{cat}\times \left({\left[\text{FtsZ}\right]}_{T}-C_{C-\text{GTPase}}\right) \end{array}$$ (2)

The catalytic rate constant for GTP hydrolysis, *k_cat_*, was calculated employing linear regression from plots of *k_obs_* vs. total protein concentration [FtsZ]_T_. The critical concentration (C_C-GTPase_) was obtained from the intercept of the linear regression with the abscissa axis.

### FtsZ polymerization kinetics

The kinetics of FtsZ polymerization were measured by 90°-angle light scattering at 1-s intervals in a Perkin Elmer LS50 fluorimeter with excitation and emission wavelengths set at 350-nm. The scattering signal was observed through a 1% transmission neutral density filter using 7-nm slits for EcFtsZ or using 4-nm slits for MjFtsZ. The temperature was controlled to ± 0.2°C using a water circulating cuvette chamber. The protein samples were incubated in polymerization buffer (the same as for the GTPase activity experiments), at the specified temperature, and the light scattering was recorded until a steady signal was reached, which usually took about 5 minutes. Then, the polymerization reaction was started by the addition of GTP to a final concentration of 1 mM.

At saturating GTP concentrations, all proteins above the critical concentration (C_C–Pol_) assemble at polymer ends and dissociate as the nucleotide is hydrolyzed. This stationary equilibrium condition may be described by (Eq 3),

$${\text{FtsZ}}_{\text{mon}}^{\text{GTP}}+{\text{FtsZ}}_{{\text{pol}}_{\left(\text{n}-1\right)}}^{\text{GTP}}\overset{K_{\text{p}}}{↔}{\text{FtsZ}}_{{\text{pol}}_{\left(\text{n}\right)}}^{\text{GTP}}\overset{hydrolysis}{\to }{\text{FtsZ}}_{{\text{pol}}_{\left(\text{n}\right)}}^{\text{GDP}}\overset{k_{depol}}{\to }n{\text{FtsZ}}_{\text{mon}}^{\text{GDP}}$$

$$\begin{array}{ccc} K_{\text{p}} & = & \frac{\left[{\text{FtsZ}}_{{\text{pol}}_{\left(\text{n}\right)}}^{\text{GTP}}\right]}{\left[{\text{FtsZ}}_{\text{mon}}^{\text{GTP}}\right]\left[{\text{FtsZ}}_{{\text{pol}}_{\left(\text{n}-1\right)}}^{\text{GTP}}\right]}\approx \frac{1}{\left[{\text{FtsZ}}_{\text{mon}}^{\text{GTP}}\right]}=\frac{1}{{\text{C}}_{\text{C}-\text{pol}}} \end{array}$$ (3)

where *K*_*p*_ is the polymer elongation equilibrium constant describing the affinity of the FtsZ-FtsZ interaction occurring at polymer ends. The superscripts GTP and GDP indicate the type nucleotide bound to the FtsZ subunits. The subscripts *mon* and *pol* represent a monomer species and a polymeric species, respectively, and *n* is the number of subunits per polymer. When the number of subunits in the polymer is large such that *n* ≫ 1, then the concentration of non-polymerized protein is the critical concentration C_C–Pol_ $\approx [{FtsZ}_{\text{mon}}^{\text{GTP}}]$. The values of C_C–Pol_ were obtained from the intersection of the linear regressions with the protein concentration axis, in plots of the extent of polymerization (Δ) *vs.* the total protein concentration [FtsZ]_T_. The extent of polymerization Δ was calculated from the difference in light scattering between the baseline and the maximal intensity recorded after addition of GTP. To determine the mean rate of polymer disassembly *k*_*depol*_ we used the light scattering data corresponding to the stage of disassembly. According to the scheme shown above, the stage of disassembly starts after the plateau when the polymers hydrolyze all the GTP available. In these conditions, the polymers bound to GDP are unstable and quickly disassemble as shown by the decrease in light scattering signal to baseline levels (see Results section for a detailed description). The onset of the stage of disassembly was considered to begin when the light scattering signal dropped below the average maximum value recorded after addition of GTP. The slope associated with the drop of light scattering signal was fit to a straight line by linear regression and was assumed to represent the mean rate of polymer disassembly. This method has been used before to measure the polymerization off-rate of tubulin and actin [31, 32].

### Calculation of the transition-state parameters Δ*G*^0‡^, Δ*H*^0‡^ and Δ*S*^0‡^

The kinetic rates of GTP hydrolysis and of depolymerization were analyzed using the Eyring equation (assuming a transmission coefficient equal to 1)(Eq 4),

$$\begin{array}{ccc} k_{obs} & = & \frac{k_{b}T}{h}exp(\frac{\Delta S^{0‡}}{R}-\frac{\Delta H^{0‡}}{RT}) \end{array}$$ (4)

where *k_obs_* is the first-order rate constant, *R* is the universal gas constant, *T* the absolute temperature, *k_b_* is the Boltzmann constant and *h* is Planck’s constant. Starting with plots of *k_obs_* *vs.* *T*, the standard enthalpy and entropy of the transition state, Δ*H*^0‡^ and Δ*S*^0‡^, were calculated using nonlinear regression directly over the experimental data using Eq 4, and assuming that both parameters are temperature-independent. The best-fit parameters were calculated employing a proportional weighting factor equal to the reciprocal of the uncertainties of each data point [33]. The standard free energy of the transition state Δ*G*^0‡^ was calculated using the relationship (Eq 5),

$$\begin{array}{ccc} \Delta G^{0‡} & = & \Delta H^{0‡}-T\Delta S^{0‡} \end{array}$$ (5)

### Calculation of the heat capacity change Δ*C*_*p*_ and the temperature-dependent parameters Δ*G*^0^, Δ*H*^0^ and Δ*S*^0^

The reciprocal of the critical concentration is, to a good approximation, equal to the apparent polymer elongation equilibrium constant, *K_p_* describing the exchange of subunits at the ends of a growing polymer. Starting from plots of *ln*(1/C_C_) *vs.* (1/*T*), the data was fit using nonlinear regression and the integrated van’t Hoff equation as follows [18, 19, 34, 35] (Eq 6):

$$\begin{array}{ccc} ln(K_{p}) & = & a+b(\frac{1}{T})+c\;ln\left(T\right) \end{array}$$ (6)

The best-fit coefficients *a*, *b* and *c* were determined using the reciprocal uncertainties of each data point as weighting factors. The values of the heat capacity change Δ*C*_*p*_, the free energy change Δ*G*^0^, the enthalpy change Δ*H*^0^ and the entropy change Δ*S*^0^, were calculated with the following equation (Eq 7):

$$\begin{array}{ccc} \Delta G^{0} & = & -RTln(K_{p}) \end{array}$$

$$\begin{array}{ccc} \Delta H^{0} & = & R(c\;T-b) \end{array}$$ (7)

$$\begin{array}{ccc} \Delta S^{0} & = & \frac{\Delta H^{0}-\Delta G^{0}}{T} \end{array}$$

$$\begin{array}{ccc} \Delta C_{p} & = & R\;c \end{array}$$

### Characterization of polymer morphology using electron microscopy

FtsZ polymerization products were negatively stained and visualized using transmission electron microscopy. The reaction mixtures were prepared as for the GTPase activity experiments and pre-warmed at the desired temperature. The polymerization of FtsZ was started by the addition of 1 mM GTP. After 30 seconds, a 10 μl sample was quickly deposited over a parafilm sheet (BEMIS, Neenah, WI), pre-warmed at the desired temperature, and incubated for additional 5 minutes. Then, a 400-mesh parlodion-coated copper grid, previously activated under UV irradiation, was placed on top of the sample drop and left untouched for 2 minutes. The loaded grids were dried over a clean filter paper and subsequently rinsed with deionized water. Immediately after, the grids were treated with 2% uranyl acetate (dissolved in water) for 2 minutes, and rinsed with deionized water to remove the excess stain and dried again. The grids were visualized in a Phillips Tecnai 12 Bio Twin Electron Microscope at 49,000X (for EcFtsZ), or with a Jeol 100 electron microscope at 20,000X (for MjFtsZ). The polymer widths were measured using ImageJ software (http://imagej.nih.gov/ij/).

## Results

### Modulation of the rates of GTP hydrolysis and depolymerization by temperature

The apparent coupling between the GTPase activity and polymerization of FtsZ provided a means for the characterization of its function by two independent experimental approaches. Initially, we examined the broadest temperature interval that allowed quantitative detection of the rates of GTP hydrolysis for both proteins at 5 μM. This examination served to establish the temperature range at which we measured FtsZ function and analyzed the thermodynamics and kinetics of the assembly process. We found that mesophilic EcFtsZ was functional in a narrower range of temperatures than thermophilic MjFtsZ. Also, the GTP hydrolysis rates of mesophilic EcFtsZ were lower than those of thermophilic MjFtsZ. More specifically, EcFtsZ showed marginal GTPase activity below 10°C and at temperatures above 30°C, we observed an inactivation process characterized by lower than expected hydrolysis rates. In the case of MjFtsZ, we observed marginal GTPase activity below 40°C and no significant inactivation was observed up to 80°C. Based on these observations we established a workable range between 10 and 30°C for EcFtsZ and between 40 and 80°C for MjFtsZ.

**Fig 1:**
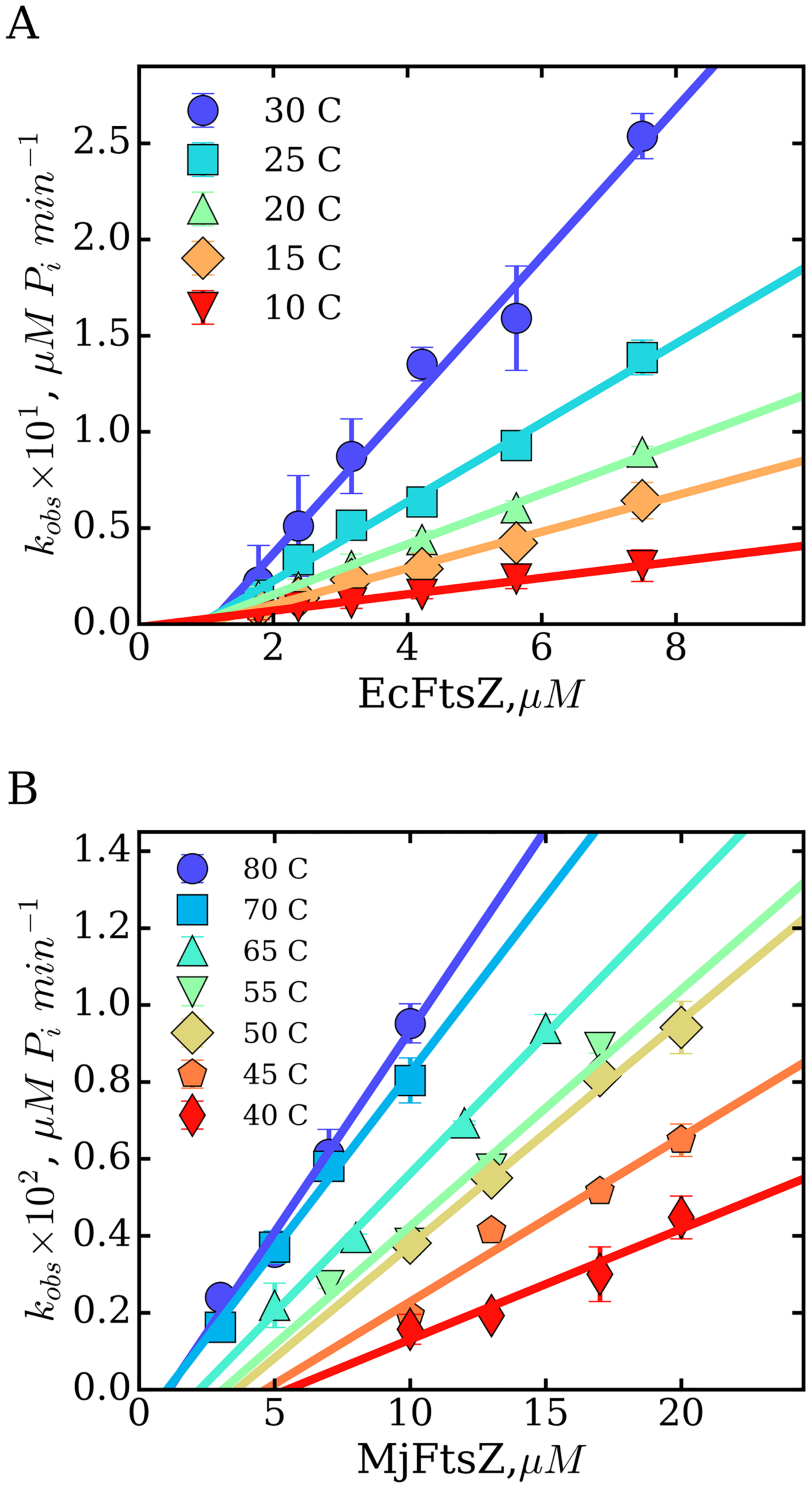
Temperature dependence of the GTP hydrolysis rates of FtsZ. The effect of temperature and protein concentration on the GTPase activity of FtsZ was examined for mesophilic EcFtsZ (A) and for thermophilic MjFtsZ (B). The catalytic constant *k_cat_* was determined from the slopes of the linear regressions indicated by the solid lines (the substrate was used at saturating concentrations). The critical concentrations C_C-GTPase_ were obtained from the intersection of the linear regressions with the protein concentration axis. The symbols and error bars are the averages and the standard deviations from triplicate samples. The calculated values of C_C-GTPase_ and *k_cat_* are presented in Tables 1 and 2.

The GTP hydrolysis rates (*k_obs_*) were determined as a function of protein concentration and plotted for the range of temperatures specified (Fig 1). A linear relationship of *k_obs_* over the total protein concentration was observed for both EcFtsZ and MjFtsZ, at all temperatures, indicating that these are first-order rate enzymatic reactions. The lack of deviations from linearity indicated that both proteins were stable throughout the experiment. Fits of the experimental data to Eq 2 allowed calculation of the rates of nucleotide turnover, or catalytic constants (*k_cat_*), from the slopes of the linear regressions (solid lines) shown in Fig 1. The calculated values of *k_cat_* are shown in Tables 1 and 2. It is evident that the rates of GTP hydrolysis *k_cat_* are enhanced by increasing the temperature, for both proteins. Since the range of temperatures examined for these proteins was different, to compare the enhancement, we determined the fold-change in *k_cat_* per 10° increase in temperature with EcFtsZ = 2.9 ± 0.7; and MjFtsZ = 1.5 ± 0.4. These values indicated a greater but comparable enhancement of the GTPase activity for mesophilic EcFtsZ over that of thermophilic MjFtsZ.

**Table 1:** Measured parameters for the GTPase activity and polymerization of FtsZ from *Escherichia coli*.

Temperature	GTPase activity		Polymerization	
°*C*	*k*_*cat*_ *s*^−1^	C_C-GTPase_ μM	*k*_*depol*_ *s*^−1^	C_C-Pol_ μM
10	24 ± 6 ^a^	0.37 ± 0.23	36 ± 6	0.18 ± 0.18
15	54 ± 6	0.70 ± 0.53	60 ± 6	0.42 ± 0.18
20	78 ± 6	0.83 ± 0.42	138 ± 6	0.64 ± 0.32
25	126 ± 7	0.91 ± 0.28	294 ± 12	0.81 ± 0.41
30	234 ± 7	1.15 ± 0.15	570 ± 18	1.10 ± 0.13

^a.^ The uncertainties are the standard errors calculated with 68% confidence.

**Table 2:** Measured parameters for the GTPase activity and polymerization of FtsZ from *Methanocaldococcus jannaschii*.

Temperature	GTPase activity		Polymerization	
°*C*	*k*_*cat*_ *s*^−1^	C_C-GTPase_ μM	*k*_*depol*_ *s*^−1^	C_C-Pol_ μM
40	174 ± 30 ^a^	5.5 ± 2.9	3.5 ± 0.2	3.9 ± 0.1
45	258 ± 36	4.7 ± 2.4	ND ^b^	ND ^b^
50	348 ± 18	3.3 ± 0.9	20 ± 2	1.8 ± 0.5
55	378 ± 42	3.3 ± 1.4	42 ± 2	ND ^b^
60	ND ^b^	ND ^b^	120 ± 2	1.6 ± 0.5
65	432 ± 18	2.2 ± 0.5	ND ^b^	ND ^b^
70	552 ± 36	1.0 ± 0.4	360 ± 23	1.6 ± 0.4
75	ND ^b^	ND ^b^	540 ± 23	ND ^b^
80	660 ± 54	1.1 ± 0.6	810 ± 25	1.1 ± 0.3

^a.^ The uncertainties are the standard errors calculated with 68% confidence.

^b.^ ND, not determined.

Having shown the stable behavior of mesophilic and thermophilic FtsZ, characterized by a linear increase of *k_obs_* as a function of protein concentration, we determined the critical concentrations for the GTPase activity (C_C-GTPase_). For both proteins, the values of C_C-GTPase_ were obtained from the intersection of the linear regressions with the protein concentration axis shown in Fig 1, and the values are shown in Tables 1 and 2. We observed that increasing temperatures induced opposite effects over C_C-GTPase_ between mesophilic and thermophilic FtsZ. While the critical concentration of EcFtsZ decreased when lowering the temperature from 30 to 10°C, inversely, the critical concentration of MjFtsZ increased when lowering the temperature from 80 to 40°C. Since the temperature intervals analyzed were different, we determined the fold-change in C_C_ induced by a 10° change in temperature, with EcFtsZ = 1.6 ± 0.4 and MjFtsZ = 1.4 ± 0.2. These values showed that despite the opposite effects of temperature over the critical concentrations determined by the GTPase activity assay, the overall magnitudes of these changes are similar for mesophilic and thermophilic FtsZ.

### Modulation of FtsZ polymerization by temperature

**Fig 2:**
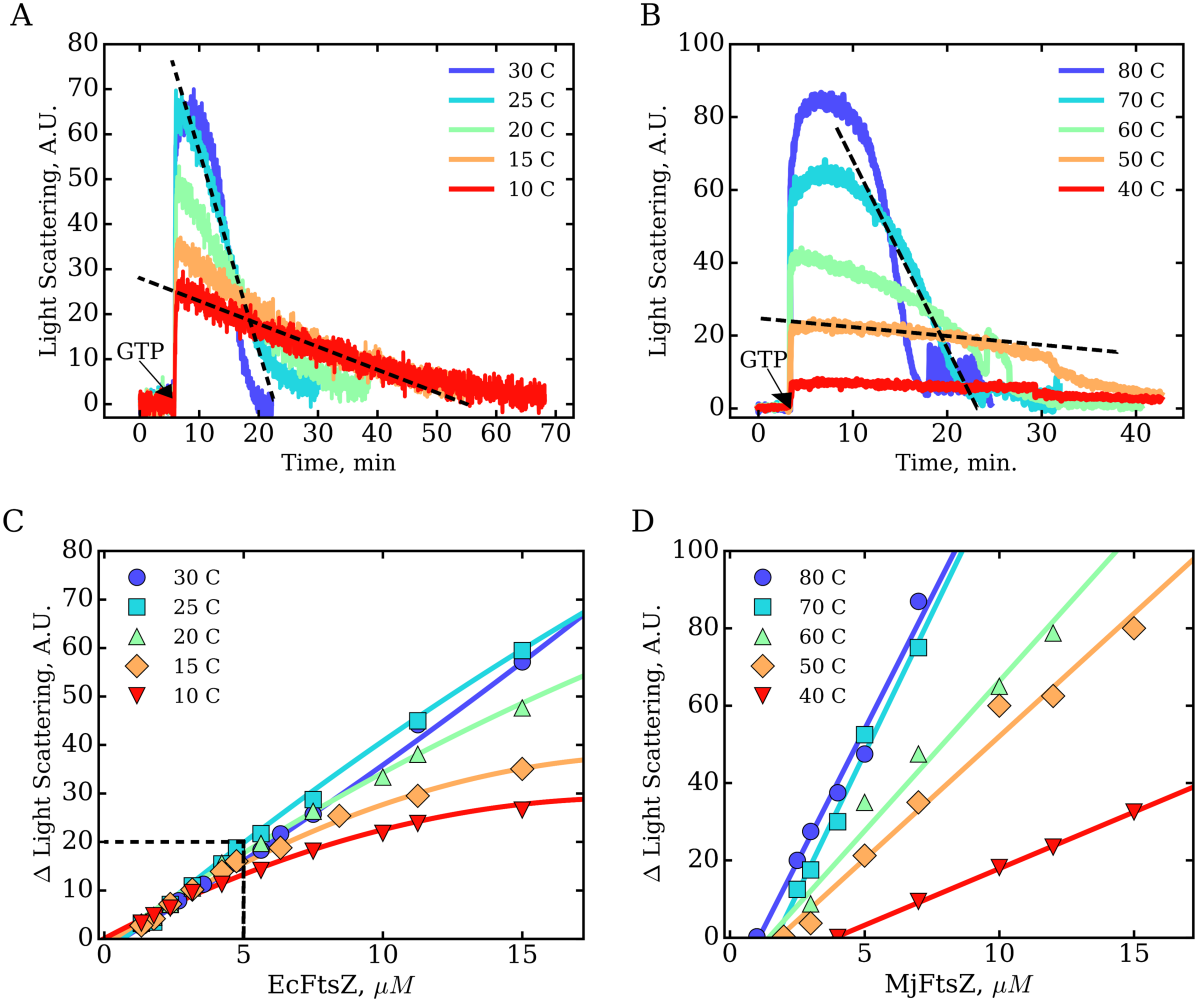
Temperature dependence of FtsZ polymerization kinetics. The effect of temperature on the polymerization kinetics was analyzed at 15 μM for mesophilic EcFtsZ (A) and at 7 μM for thermophilic MjFtsZ (B). The depolymerization rates *k_depol_* were determined by calculating the slope of the kinetic traces representing the disassembly stage (dotted lines). The extent of polymerization was obtained from the difference in light scattering Δ between the baseline and the maximal signal obtained after addition of GTP (indicated by the arrow), and was plotted as a function of concentration for mesophilic EcFtsZ (C) and for thermophilic MjFtsZ (D). At each temperature, the critical concentration C_C–Pol_ was obtained from the intersection of the linear regression with the protein concentration axis (see Materials and methods). In panel C, the solid lines show the trends of the data, and the linear regression analysis was done with data at concentrations below 5 μM (indicated by the dashed box). The calculated values are reported in Tables 1 and 2.

To study the effect of temperature on the assembly of FtsZ we measured the kinetics of polymerization using light scattering. We determined the depolymerization rates *k_depol_* and the critical concentrations for polymerization C_C–Pol_. These two quantities are each related to the previously described *k_cat_* and C_C–GTPase_, respectively. The kinetic traces shown in Fig 2A and 2B, indicate that the polymerization reaction can be divided into three stages. First, a sharp increase in the light scattering signal was observed after addition of the GTP nucleotide, indicating a fast on-rate for the polymerization reaction. Second, a short stationary polymerized state follows the onset of assembly which was clearly influenced by changing the temperature, for both mesophilic EcFtsZ and thermophilic MjFtsZ. The third stage, representing disassembly of FtsZ bound to GDP, also influenced by temperature, was used for calculation of *k_depol_* with values reported in Tables 1 and 2 (see Materials and methods). We observed an enhancement in *k_depol_* with increasing temperatures for both proteins. Since the temperature interval examined was different between the two FtsZ orthologs, we quantified the fold-change in *k_depol_* per 10° increase in temperature, with EcFtsZ = 4.3 ± 0.5 and MjFtsZ = 4.2 ± 1.6. These values indicate that the mean rates of disassembly for EcFtsZ and MjFtsZ, are similarly modulated by temperature.

The critical concentrations for polymerization C_C–Pol_ were obtained from the dependence of the extent of polymerization, Δ, with the protein concentration shown in Fig 2C and 2D. For both mesophilic and thermophilic FtsZ, we observed an enhancement of Δ with increasing temperatures. However, the relationship of Δ with the protein concentration was different for both FtsZ orthologs. The extent of polymerization Δ for thermophilic MjFtsZ followed a linear relationship with the protein concentration and the critical concentration C_C–Pol_ was determined by linear regression (Fig 2D). In contrast, the extent of polymerization Δ for mesophilic EcFtsZ showed a more complex behavior that was linear at 30 and 25°C but showed a downward curvature at lower temperatures, and at EcFtsZ concentrations above 5 μM (Fig 2C). These results suggested that by lowering the temperature, the EcFtsZ polymers adopted a shape that scattered light differently (see below). Despite this, below 5 μM EcFtsZ, the extent of polymerization Δ followed a linear relationship with the protein concentration (indicated by a dashed square in Fig 2C). We therefore obtained the critical concentrations of EcFtsZ by linear regression, using these data at concentrations below 5 μM. The resulting values of the critical concentrations are shown in Tables 1 and 2. We observed that lowering the temperature induced opposite effects over C_C–Pol_ between the mesophilic and thermophilic FtsZ. While the critical concentration of EcFtsZ decreased when lowering the temperature from 30 to 10°C, inversely, the critical concentration of MjFtsZ increased when lowering the temperature from 80 to 40°C. This behavior of C_C–Pol_ over temperature was consistent with that previously described for C_C–GTPase_. Since the temperature interval analyzed was different, we determined the fold-change in C_C_ induced by a 10± change in temperature with EcFtsZ = 2.3 ± 0.8 and MjFtsZ = 1.5 ± 0.4. These values indicated a greater, but comparable, change of C_C–Pol_ between mesophilic and thermophilic FtsZ.

### Modulation of FtsZ polymer morphology by temperature

**Fig 3:**
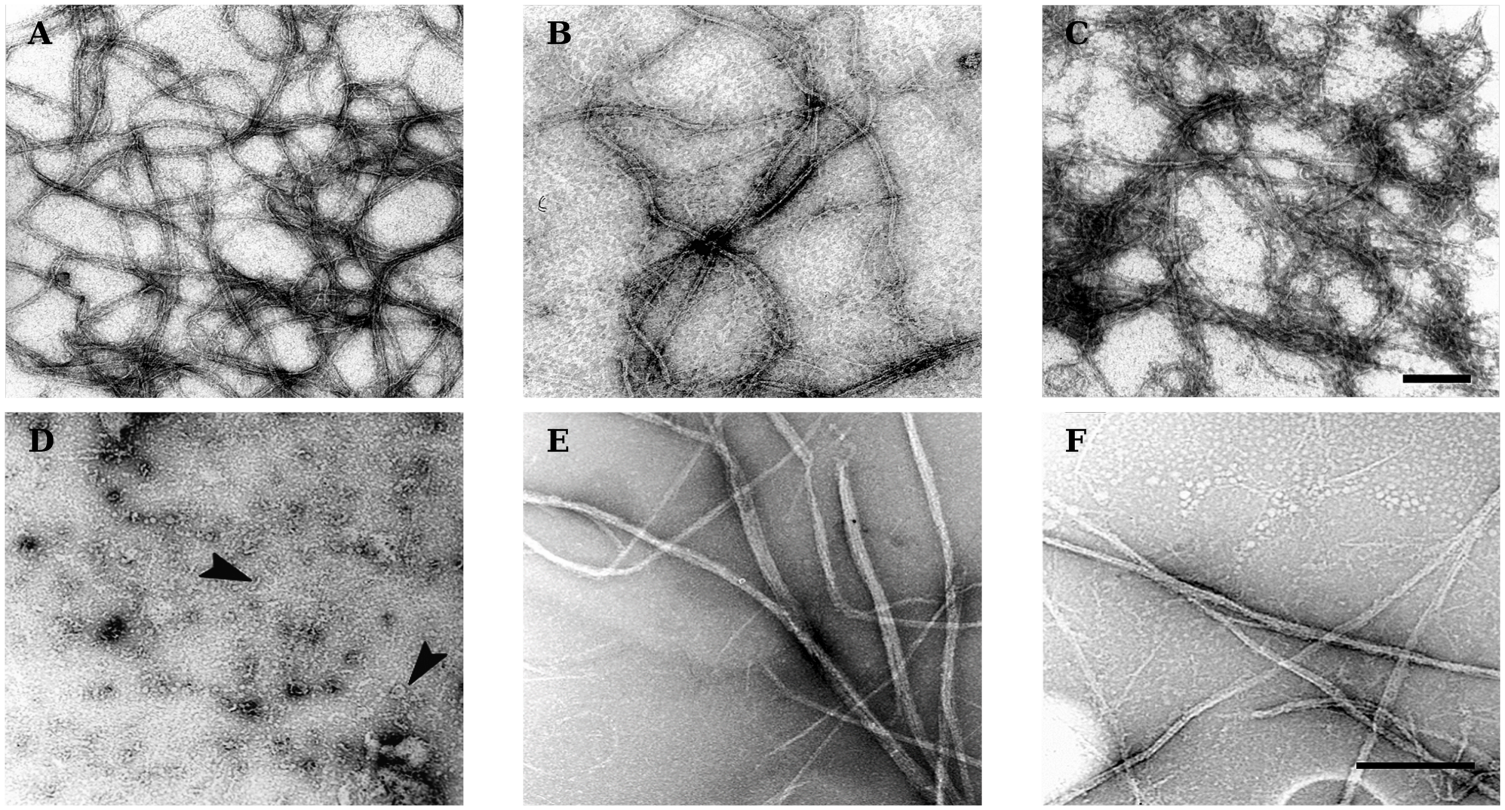
The morphology of FtsZ polymers was characterized by negative-stain transmission electron microscopy. Top row, 10 μM EcFtsZ was polymerized at 10, 20 and 30°C (A-C). Bottom row, 10 μM MjFtsZ was polymerized at 40, 60 and 80°C (D–F). The arrowheads in D point to examples of short and curved polymers. The scale bars represent 100 and 500 nm for the top and bottom rows, respectively (black bars).

We observed the polymers formed by both FtsZ orthologs at various temperatures using electron microscopy (Fig 3). Polymers longer than 500 nm (>120 subunits) were observed in samples of both proteins (Fig 3C and 3F). At 30°C, the polymers formed by mesophilic EcFtsZ appeared curved as if they were flexible. In contrast at 80°C, MjFtsZ polymers were straight as if they were more rigid. Also, while EcFtsZ polymers were predominantly single- or double-stranded, MjFtsZ polymers appeared as bundles comprising up to ten protofilaments. We found that mesophilic EcFtsZ polymerized at the low temperature of 10°C forming filaments of normal aspect (Fig 3A). This result indicated that the downward curvature observed in Fig 2C, for the extent of polymerization Δ of EcFtsZ, was not related to a lower degree of polymerization but to more complex optical effects that we cannot explain with the current data. In the case of thermophilic MjFtsZ, the polymers appeared very short and curved in the sample incubated at 40°C, which was the lowest temperature at which we could detect a signal increase indicative of polymerization (Fig 2B). These data show that while EcFtsZ readily polymerized at low temperatures, in the case of MjFtsZ, polymerization at low temperatures produced short and curved filaments that are not related to those observed at higher temperatures.

### Transition state analysis of FtsZ assembly

**Fig 4:**
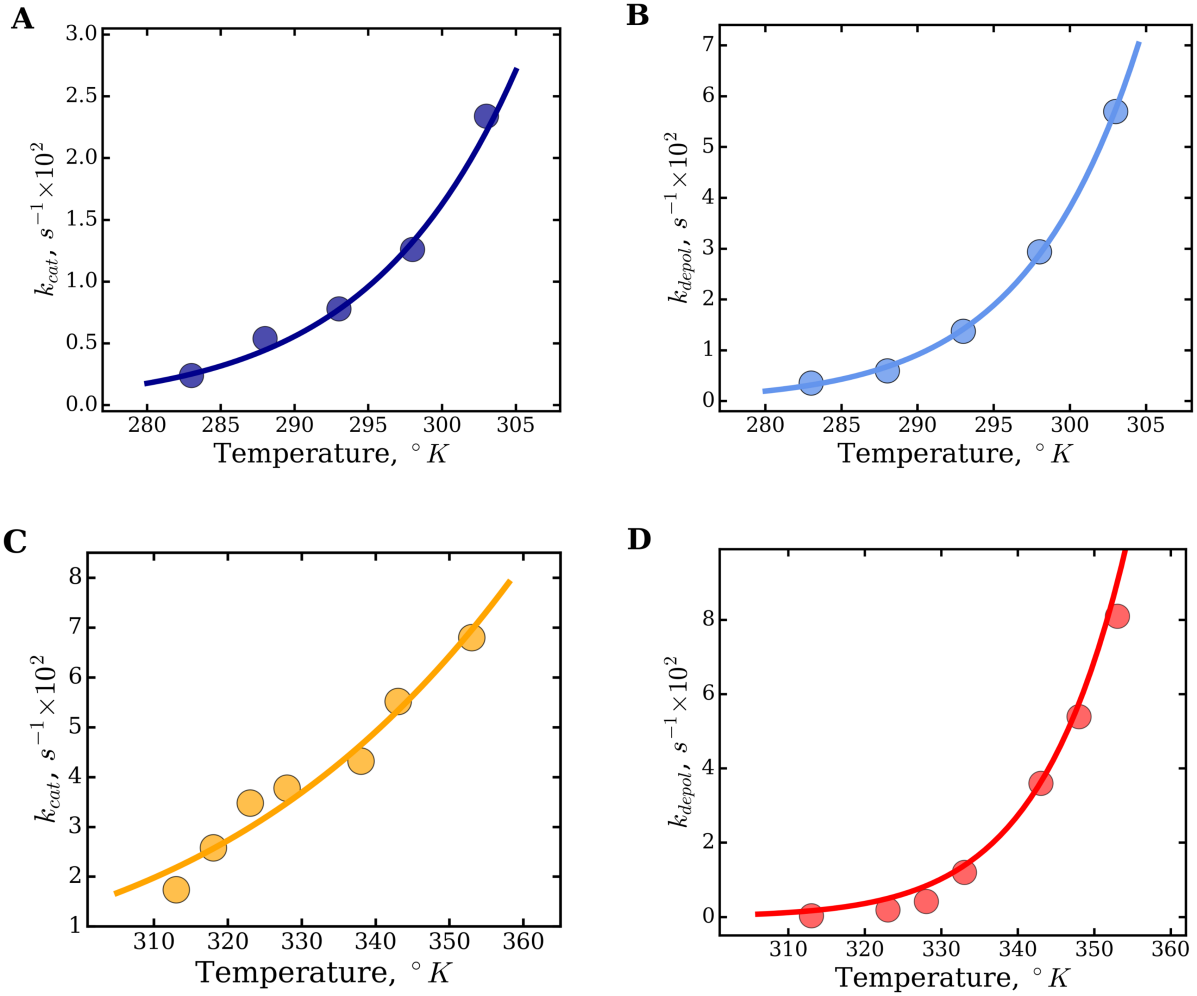
Eyring plots of the GTPase activity and polymerization of FtsZ. Top row, mesophilic EcFtsZ. Bottom row, thermophilic MjFtsZ. The kinetic rates of GTP hydrolysis, *k_cat_* (A and C), and filament depolymerization, *k_depol_* (B and D), are plotted as a function of temperature. The apparent enthalpies (Δ*H*^0‡^) and entropies (Δ*S*^0‡^) of the transition state were calculated by nonlinear regression using Eq 4 (solid lines), and the best-fit values are reported in Table 3.

To investigate the relative contributions of enthalpy and entropy to the stability of the [FtsZ − GTP]^‡^ transition-state complex we modeled the rates of nucleotide turnover *k_cat_*, and the independently determined depolymerization rates *k_depol_*, using Eyring’s transition state theory (Fig 4). In the range of temperatures analyzed, the Eyring plots showed a clear increase of the kinetic rates with temperature, for both mesophilic EcFtsZ and thermophilic MjFtsZ. The transition state enthalpy (Δ*H*^0‡^) and entropy (Δ*S*^0‡^) were calculated with nonlinear regression using Eq 4 (solid lines in Fig 4), and the resulting values are reported in Table 3. We observed positive values of Δ*H*^0‡^ across all four data sets, indicating that destabilization of the transition state complex [FtsZ − GTP]^‡^ is an endothermic process. In three cases the entropy Δ*S*^0‡^ was positive suggesting that destabilization of the transition state complex is spontaneous and entropically-driven. For the particular case of the GTP hydrolysis rates of MjFtsZ, the entropy was negative, suggesting that destabilization of the transition state complex is forbidden. Which is obviously not the case, since the protein does show GTP hydrolysis and also depolymerizes after a while. Visual inspection of the Eyring plot in Fig 4C showed a rather shallow curve of the *k_cat_* data over the temperature coordinate indicating a low degree of cooperativity for this reaction. The standard free energy of the transition state Δ*G*^0‡^ was calculated from the best-fit values of Δ*H*^0‡^ and Δ*S*^0‡^ using Eq 5), resulting in consistent (and positive) values across all four data sets. Thus, the kinetic stability of the [FtsZ − GTP]^‡^ transition state complex is similar in magnitude for both mesophilic and thermophilic FtsZ.

**Table 3:** Transition state enthalpy, entropy and free energy of FtsZ assembly.

		Δ*H*^0‡^ ^a^ *kJmol*^−1^	Δ*S*^0‡^ ^a^ *JK*^−1^ *mol*^−1^	Δ*G*^0‡^ ^b^ *kJmol*^−1^
*EcFtsZ*	*GTPase*	78.4 ± 5.4	58.9 ± 1.8	61.2 ± 1.5
	*Depolymerization*	98.7 ± 1.9	134 ± 6.2	61.0 ± 1.4
*MjFtsZ*	*GTPase*	21.7 ± 1.2	-131 ± 3.5	64.8 ± 1.9
	*Depolymerization*	79.2 ± 0.8	33.8 ± 2.4	67.9 ± 1.5

^a.^ Enthalpies and Entropies of the transition state were obtained with nonlinear regression from plots shown in Fig 4. Values are the best-fits ± standard errors calculated with 68% confidence.

^b.^ The free energy of the transition state was calculated using the relationship Δ*G*^0‡^ = Δ*H*^0‡^−*T*Δ*S*^0‡^ and averaged across all temperatures.

### Thermodynamic analysis of FtsZ assembly

**Fig 5:**
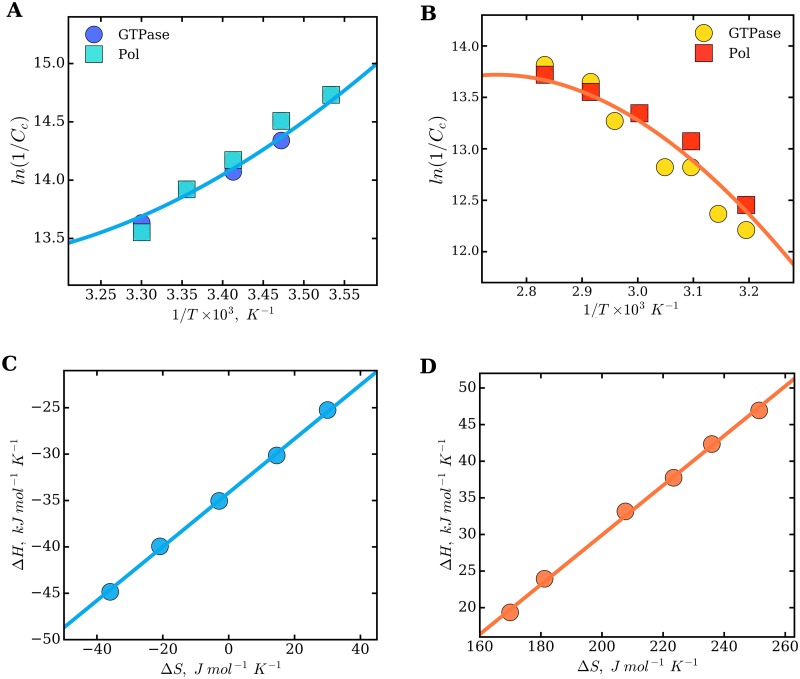
Global analysis of the critical concentration using the integrated van′t Hoff equation. The combined data obtained with the GTP hydrolysis and polymerization assays were fit to Eq 6 using nonlinear regression (solid lines), for mesophilic EcFtsZ (A) and thermophilic MjFtsZ (B). The fitting coefficients for EcFtsZ are: *a* = -787.59, *b* = 38,745 and *c* = 117.86, and the coefficients obtained for MjFtsZ: *a* = 776.24, *b* = -40,253 and *c* = -110.53. The temperature-dependent parameters Δ*H*^0^ and Δ*S*^0^ were calculated using Eq 7 (Panels C and D), as well as the temperature-independent heat capacity change Δ*C*_*p*_ (see text).

The assembly of thermophilic MjFtsZ has been described as an endothermic and entropically driven process [19]. The thermodynamics of mesophilic EcFtsZ function have not been determined before and presumably are similar to those of mesophilic MjFtsZ. In this work, the modulation of FtsZ function by temperature was examined for both mesophilic and thermophilic FtsZ. Visual inspection of the van’t Hoff plots shown in Fig 5, *ln*(1/C_C_) *vs.* (1/*T*), showed a curvature and opposite behaviors between EcFtsZ and MjFtsZ. Previous studies described this curvature in the van’t Hoff plots as due to a nonzero heat capacity change (Δ*C*_*p*_ ≠ 0) for the elongation reaction. The thermodynamic parameters Δ*G*^0^, Δ*H*^0^, Δ*S*^0^ and Δ*C*_*p*_ were calculated using an integrated form of the van’t Hoff equation [19, 34, 35]. Here, we analyzed the combined data obtained from the GTP hydrolysis and polymerization experiments, by global analysis, using the integrated van’t Hoff equation (Eq 6) (solid lines in Fig 5A and 5B). The relationship between the enthalpies and entropies of elongation, calculated with Eq 7, are shown in Fig 5C and 5D, for mesophilic EcFtsZ and thermophilic MjFtsZ, respectively. By considering the thermodynamic parameters determined using the integrated van’t Hoff equation (extrapolated to the optimal growth temperature), the assembly of mesophilic EcFtsZ is characterized by a negative enthalpy (-18.4 *kJmol*^−1^), a positive entropy (44.4 *JK*^−1^ *mol*^−1^), and by a positive heat capacity change: Δ*C*_*p*−*EcFtsZ*_ = 979 ± 687*JK*^−1^ *mol*^−1^. The assembly of thermophilic MjFtsZ is characterized by a positive enthalpy (5.57 *kJmol*^−1^) and a positive entropy (132 *JK*^−1^ *mol*^−1^), and the heat capacity change is negative: Δ*C*_*p*−*MjFtsZ*_ = -920 ± 229*JK*^−1^ *mol*^−1^.

## Discussion and conclusions

The GTP hydrolysis and polymerization of FtsZ are both manifestations of the reversible association of the FtsZ subunits. Based on previous studies of the energetics of thermophilic MjFtsZ assembly, we hypothesized that the assembly of mesophilic EcFtsZ may be similarly modulated by temperature. To test this prediction, we measured the rates of GTP hydrolysis (and of depolymerization) and the critical concentrations of FtsZ, over a range of temperatures close to the physiological temperatures of mesophilic *Escherichia coli* (∼37°C) and of thermophilic *Methanocaldococcus jannaschii* (∼85°C). We analyzed the kinetics and thermodynamics of FtsZ assembly and compared our observations with previous data about the function of thermophilic FtsZ and of eukaryotic tubulin.

### Entropy-driven destabilization of the [FtsZ − GTP]^‡^ transition state complex

The kinetics of FtsZ disassembly were analyzed using Eyring’s transition state theory to calculate the enthalpy Δ*H*^0‡^ and entropy Δ*S*^0‡^ for the destabilization of the transition state complex [FtsZ − GTP]^‡^. This intermediate state is hypothesized to be FtsZ subunits bound to GTP that are incorporated in the polymers, before disassembly is promoted by the hydrolysis of the nucleotide. We could fit the data well by using a single-component model, indicating that the disassembly reaction may be described by a first-order rate law. Overall, we observed similar activation parameters for the assembly of mesophilic EcFtsZ in comparison with those of thermophilic MjFtsZ. For instance, the transition state enthalpies were relatively large and positive for the two FtsZ orthologs, indicating an endothermic destabilization of the [FtsZ − GTP]^‡^ complex. This finding is consistent with higher rates of GTP hydrolysis at increasing temperatures, as predicted by transition state theory. With one exception, we observed a positive entropy of activation, indicating an entropy-driven destabilization of the [FtsZ − GTP]^‡^ complex. The exceptional case, with a negative entropy, is that of the GTP hydrolysis rates for thermophilic MjFtsZ. In the other three cases the activation entropies were positive. This difference can be understood if we consider the morphology of the polymers formed by MjFtsZ. We observed a number of tubular polymer bundles in the micrographs of MjFtsZ, that appeared to contain several laterally-associated protofilaments. We considered the hypothetical scenario where the FtsZ subunits that assembled into these tubular bundles are confined, and therefore the exchange and diffusion of the subunits (and of the bound nucleotide) is limited. This scenario could explain a negative entropy observed for the GTP-hydrolysis assay of MjFtsZ. In our interpretation of a positive entropy for destabilization of the [FtsZ − GTP]^‡^ transition-state complex, the primary source of entropy increase comes from the disassembly of monomers from polymer ends, in other words, the passage from a fixed state in the protofilament to a freely diffusing monomeric state in the bulk of the solution. The other factor contributing to the observed entropy increase is the release of the hydrolyzed P_i_ along the protofilaments which subsequently diffuses into the bulk of the solution.

Since there are no previous data on the thermodynamics of activation for FtsZ, we compared our findings with the data reported for tubulin and actin polymerization. In this work, we characterized the rates of depolymerization *k_-1_* which were readily measurable under our experimental conditions. The polymerization on-rates *k_+1_* were too fast to be measurable, for both mesophilic EcFtsZ and thermophilic MjFtsZ. In the case of tubulin, both rates have been measured as a function of temperature from which the activation enthalpies and entropies were determined, which were both positive [31, 36]. However, for the depolymerization rates of tubulin, a disagreement was encountered in the sign of the enthalpy of activation, where both positive and negative values were obtained depending on the range of temperatures analyzed. These discrepancies resulted in a biphasic shape of the Arrhenius plots (or Eyring plots) of tubulin depolymerization [31]. When the assembly of tubulin was analyzed in the presence of paclitaxel, the overall activation enthalpy and entropy were both found to be positive [37]. In the case of actin (an ATPase), which polymerizes forming helical-double-stranded filaments, a closer shape to FtsZ double filaments, the activation enthalpy and entropy were both found to be positive [32]. Hence, the activation parameters found in this work for the assembly of mesophilic EcFtsZ and thermophilic MjFtsZ, are in good agreement with the data reported for these related reversibly-polymerizing proteins. We may propose a mechanism where the transition state complex of FtsZ, and of other polymerizing proteins such as tubulin and actin, is destabilized by the release of protein subunits from polymer ends, and the exchange of GDP/GTP nucleotides after hydrolysis, both phenomena resulting in a positive entropy for the depopulation of transition-state complex.

### Thermodynamics of assembly of mesophilic EcFtsZ differ from that of thermophilic MjFtsZ

To analyze the thermodynamic data of mesophilic and thermophilic FtsZ assembly we used an integrated form of the van′t Hoff equation (Eq 6). Using this approach, we calculated Δ*G*^0^, Δ*H*^0^, Δ*S*^0^ and Δ*C*_*p*_ for the elongation reaction. Of the four thermodynamic parameters we determined for the assembly of FtsZ, the latter has been regarded as the richest in information but the hardest to understand [38](see below for a discussion). In this study, it was assumed that the apparent association constant describing the addition of a FtsZ monomer to the end of a growing protofilament is equal to the reciprocal of the critical concentration [34]. The Gibbs free energy is by definition related to the equilibrium elongation constant, and thereby also related to the critical concentrations obtained directly from our experiments. However, care must be taken in interpreting the thermodynamic data obtained from measurements of the critical concentration. The hypothesized steady-state equilibrium attained after addition of the nucleotide is not a bona fide reversible equilibrium [39], because is mediated by the hydrolysis of GTP, and the values of *K_p_* = 1/C_C_ are an average of the assembly-disassembly events occurring at both filaments ends. Moreover, the free energy determined using this parameter also carries the contribution of any additional interaction established by FtsZ subunits, such as lateral associations. For these reasons, C_C_ is not considered a system parameter. Nonetheless it provides a useful parametrization of polymer growth, of which the association of monomers to the growing polymer is a major component.

The function of FtsZ has been reported over a range of temperatures. In the case of FtsZ from *E. coli* (and from other similar mesophilic organisms) the temperatures used for in vitro studies ranged from 25 to 37°C [40–45]. In the case of FtsZ from *M. jannaschii* the predominant temperature used was 55°C [16, 45–49]. A critical concentration of 1 μM has been proposed for FtsZ polymerization in general [26], but in the literature the numbers vary from 0.1 to 3 μM [18, 28, 50]. Our calculated values for the Gibbs free energy are indeed similar between mesophilic EcFtsZ and thermophilic MjFtsZ, as expected from the information currently available in the literature. By considering that Δ*H*^0^ and Δ*S*^0^ vary with temperature, as is assumed in the analysis using the integrated van’t Hoff equation, we calculated the associated Δ*C*_*p*_ from the curvature of the plot. Using a global nonlinear regression analysis of the combined critical concentration data, we calculated the temperature-dependent Δ*H*^0^ and Δ*S*^0^. The calculated heat capacity changes were: Δ*C*_*p*−*EcFtsZ*_ = 979 ± 687*JK*^−1^ *mol*^−1^ and Δ*C*_*p*−*MjFtsZ*_ = -920 ± 229*JK*^−1^ *mol*^−1^. The thermodynamics of the assembly of thermophilic MjFtsZ and of eukaryotic tubulin have been studied before and in both cases showed a downward curvature of the van’t Hoff plots, indicating a negative change in heat capacity [19, 34, 35]. The reported values are: Δ*C*_*p*−*Mj*_ = -3,296 *JK*^−1^ *mol*^−1^ and Δ*C*_*p*−*tubulin*_ = -6,276 *JK*^−1^ *mol*^−1^ which are both negative as is the value calculated in this work for thermophilic MjFtsZ. To date no studies of the thermodynamics of mesophilic EcFtsZ assembly have been reported.

The change in heat capacity produced by the interaction of biological macromolecules has been regarded as the major contributor to the thermodynamics of association. Earlier studies provided the basis for establishing a quantitative relationship between Δ*C*_*p*_ in units of *JK*^−1^ *mol*^−1^, and the properties of the surface of interaction as: Δ*C*_*p*_ = *C*_*np*_ ⋅ Δ*A*_*np*_ + *C*_*p*_ ⋅ Δ*A*_*p*_, whereΔ*A* is the change in nonpolar and polar solvent accessible surface area (ASA), respectively, and *C*_*p*/*np*_ ′area coefficient for hydration heat capacity′ having units of *JK*^−1^ *mol*^−1^Å^–2^ [38, 51–53]. The sign and magnitude of these coefficients vary for different types of macromolecules involved in the reaction. Model compound data shows opposite contributions of polar and nonpolar surface to the overall Δ*C*_*p*_ [38]. Then, our data suggests that for mesophilic EcFtsZ the formation of polymers is accompanied by a greater burial of polar surface residues, whereas in the case of thermophilic MjFtsZ the formation of FtsZ-FtsZ contacts is accompanied by a greater burial of nonpolar surface residues. By consideration of the structural data available for FtsZ, where the FtsZ-FtsZ interaction interface can be recognized [16], we determined the total solvent-accessible surface area of the protofilament interface for MjFtsZ (from the crystal structure) and for EcFtsZ (from an homology model), resulting in ∼2,700 Å^–2^ for both FtsZ orthologs. Also, in both cases the fraction of nonpolar and polar (including charged) residues in the interface are *f_np_* = 0.48 and *f_np_* = 0.52, respectively. These numbers show that the fraction of surface area buried upon formation of the FtsZ-FtsZ interaction is approximately equally composed of polar and nonpolar residues, for both FtsZ orthologs. In light of these data, the interpretation of Δ*C*_*p*_ based on models of burial of surface area only partially explain the differences observed between the thermodynamics of assembly of mesophilic and thermophilic FtsZ. Presumably, the surface of interaction may be affected by other processes, such as conformational transitions, that alter the geometry of the surface changing the base affinity of the interaction. It is tempting to propose that the more rigid structure of thermophilic MjFtsZ would not be subject to such conformational transitions as in the case of the more flexible mesophilic EcFtsZ, resulting in differences of the heat capacity for the elongation reaction.

### FtsZ adapted to assemble in cold and hot environments by optimization of the critical concentration

In this work, we observed that the critical concentrations for the assembly of mesophilic and thermophilic FtsZ had a similar value (1–2 μM), at temperatures close to their normal growth temperature of 37 and 85°C, respectively. Previously, the same was found for eukaryotic tubulin from Antarctic fish which assembles into microtubules with a C_C_ at 0°C (∼1 mg/mL) that is similar to C_C_ of mammalian tubulin at 37°C (∼2 mg/mL) [39, 54]. Therefore the mechanism of adaptation to different environments of eukaryotic tubulin involved the modulation of the ability of the dimers to associate with similar affinities at these different temperatures [39, 55]. Tubulin cold adaptation was found to correlate with residue substitutions in structural elements participating in the lateral interaction as well as in the core of the tubulin subunit. Here, we found that mesophilic EcFtsZ forms stable polymers at 10°C when compared to thermophilic MjFtsZ, which formed atypical short polymers at 40°C. This observation means that mesophilic EcFtsZ shows a relatively higher affinity for association at low temperatures, as was observed for Antarctic fish tubulin (psychrophilic), in other words, these proteins form cold-stable polymers. In contrast, thermophilic MjFtsZ and mammalian tubulin (mesophilic) both produce unstable polymers at lower than physiological temperatures. Accordingly, the adaptation of FtsZ is similar to that of tubulin in that the critical concentration for polymerization is conserved in mesophilic and thermophilic variants. Taken together, the data presented here and previous studies on the energetics of assembly by members of the FtsZ/Tubulin family of GTPases, indicate a conserved mechanism of adaptation by modulation of the critical concentration.
